# Origin and demographic history of the endemic Taiwan spruce (*Picea morrisonicola*)

**DOI:** 10.1002/ece3.698

**Published:** 2013-08-15

**Authors:** Sofia Bodare, Michael Stocks, Jeng-Chuann Yang, Martin Lascoux

**Affiliations:** 1 Department of Ecology and Genetics, Evolutionary Biology Centre, Uppsala UniversityUppsala, Sweden; 2 Botanical Garden Division, Taiwan Forestry Research InstituteTaipei, Taiwan

**Keywords:** ABC, bottleneck, nucleotide diversity, *Picea morrisonicola*, power, Quaternary, Taiwan

## Abstract

Taiwan spruce (*Picea morrisonicola*) is a vulnerable conifer species endemic to the island of Taiwan. A warming climate and competition from subtropical tree species has limited the range of Taiwan spruce to the higher altitudes of the island. Using seeds sampled from an area in the central mountain range of Taiwan, 15 nuclear loci were sequenced in order to measure genetic variation and to assess the long-term genetic stability of the species. Genetic diversity is low and comparable to other spruce species with limited ranges such as *Picea breweriana*, *Picea chihuahuana,* and *Picea schrenkiana*. Importantly, analysis using approximate Bayesian computation (ABC) provides evidence for a drastic decline in the effective population size approximately 0.3–0.5 million years ago (mya). We used simulations to show that this is unlikely to be a false-positive result due to the limited sample used here. To investigate the phylogenetic origin of Taiwan spruce, additional sequencing was performed in the Chinese spruce *Picea wilsonii* and combined with previously published data for three other mainland China species, *Picea purpurea, Picea likiangensis,* and *P. schrenkiana*. Analysis of population structure revealed that *P. morrisonicola* clusters most closely with *P. wilsonii,* and coalescent analyses using the program MIMAR dated the split to 4–8 mya, coincidental to the formation of Taiwan. Considering the population decrease that occurred after the split, however, led to a much more recent origin.

## Introduction

In the face of global warming, many species will either have to adapt to the new conditions, migrate to new suitable areas, or go extinct. For some species, global warming will be a greater challenge than for others. For example, species confined to islands or other isolated geographic areas will have limited opportunities to migrate to new areas. The very same species will, due to their restricted geographic distribution range, often have small effective population sizes and therefore have a limited ability to adapt to the new climate. An example of such a species is the Taiwan endemic (*Picea morrisonicola* Hay.), which occurs in a very restricted natural distribution range and is listed as “Vulnerable” by the International Union for Conservation of Nature and Natural Resources (IUCN) (Zhang et al. 2013) due to overexploitation from logging.

As the southernmost spruce species (The Gymnosperm Database, http://conifers.org/pi/Picea.php), the Taiwan spruce is subjected to very different climatic pressures than those experienced by its mainland or boreal relatives. During Pleistocene (2.6 mya to 11,700 years ago), repeated climatic oscillations took place. Pollen analyses indicate that high-altitude species, such as *Tsuga chinensis*, *Abies kawakamii,* and *P. morrisonicola* occupied lowland areas of central Taiwan between 60,000 and 50,000 years ago when the climate was cooler than today (Tsukada 1966). However, the climate in Taiwan has steadily warmed over the last 60,000 years and the increasing temperature has meant that expanding populations of both temperate and subtropical species have pushed *P. morrisonicola* into the higher altitudes of the island (Tsukada 1966). As such, *P. morrisonicola* represents an example of a species that has already had to adapt to a warming climate, intense competition, and a restricted area into which to escape. It is therefore of interest to study how these circumstances have shaped present and historical levels of genetic variation so that we can understand the impact of climate change. A great deal can therefore be learned by studying the impact that these climate shifts have had on genetic variation.

Taiwan provides in itself an intriguing setting for population genetic studies. Proto-Taiwan was formed by a collision of the Eurasian and the Philippine Sea plates likely about 9 mya, although estimates vary. This was followed by a period of tectonic and volcanic activity before it acquired its modern shape (Sibuet and Hsu 1997, 2004). Since then, it has intermittently been connected to mainland China via a land bridge during glacial maxima, enabling it to serve as a refugium. The floristic relationship between Taiwan and mainland China is probably complex. While some studies have found close resemblance and recent speciation processes, 25% of Taiwan's vascular flora is endemic. Also, Taiwan has been found to harbor plant species of diverse origins, from geographically close areas in southeastern China to the more distant tropical Asia and even temperate regions (Hsieh 2002; Chiang and Schaal 2006). Conifer species have been present on Taiwan for at least a few million years, as seen in *Taxus* (Gao et al. 2007) and *Taiwania* (Chou et al. 2011), which colonized the island 1.1 and 3.3 mya, respectively. Although several studies have been published on the floristic relationships between Taiwan and mainland China, a lot remains to be done.

There are therefore three main aims that can be pursued. Firstly, we sequenced 15 nuclear loci in 15 individuals from a natural population of *P. morrisonicola* to produce what is, to the best of our knowledge, the first population genetic study in Taiwan spruce that looks at multiple nuclear loci. Genetic data from chloroplast and mitochondrial DNA for *P. morrisonicola* have previously only focused on phylogenetic or comparative studies covering the genus *Picea* (Bouillé et al. 2010; Ran et al. 2006) or the family Pinaceae (Lin et al. 2010). Using this population genetic data set we can therefore investigate whether Taiwan spruce harbors similar levels of genetic diversity to other spruce species with similarly restricted distributions.

Secondly, by capturing within-species genetic diversity, we assess the impact that a changing climate has had on the effective population size through time by testing a null model of constant effective population size against alternative demographic models. Although the sample is small in this study, it may be sufficient as sampling of multiple independent loci is more informative about the ancestral process in comparison to sampling more individuals. However, given that the extent of sampling does impact the ability to distinguish between competing demographic models, for example, very recent population growth (e.g., Keinan and Clark 2012), we simulate data sets under a number of different models and assess the power and false-positive rate of model choice in ABC for a sample of our size.

Third, we compare sequence data for a subset of these genes in four spruce species from mainland China, *Picea likiangensis*, *Picea purpurea*, *Picea schrenkiana,* and *Picea wilsonii*, which we had already sequenced previously (Li et al. 2010). These species represent the main clades from which *P. morrisonicola* could derive (Ran et al. 2006). In total, 12 Asian spruce species have been assigned to two clusters, where *P. morrisonicola* has a basal position within the *P. wilsonii/P. purpurea* clade. Furthermore, *P. morrisonicola* has been found to be a close relative to *P. wilsonii* in a multilocus study of the complete *Picea* genus (J. Liu, pers. comm.). Hence, these four species provide a basis for estimating divergence time and gene flow between *P. morrisonicola* and species from mainland China. Specifically, we use two complementary Bayesian methods, approximate Bayesian computation (ABC; Beaumont et al. 2002) and MIMAR (Becquet and Przeworski 2007) to estimate parameters from between-species coalescent models. While both methods use coalescent simulations to model the ancestral process, each method has its own strengths and weaknesses. ABC compares summary statistics calculated from the data with those simulated under a model specified by the user. This method assumes that the summary statistics chosen sufficiently capture aspects of the data relevant to the model. However, it is efficient and flexible to the extent that a number of different models can be tested and compared with one another. Contrastingly, MIMAR is a Markov chain Monte Carlo method that estimates parameters under an isolation-with-migration (IM) model. Although it is less time efficient than ABC, it has been shown to reliably estimate the parameters of an IM model under a number of simplifying assumptions (Becquet and Przeworski 2007, 2009). We therefore seek to utilize the strengths of each method by performing model choice in ABC and comparing parameter estimates from both MIMAR and ABC to strengthen our conclusions. We complement these approaches with the clustering algorithm implemented in *Structure* (Pritchard et al. 2000) to assess the relationship of this endemic species with those from mainland China.

## Materials and Methods

### Data collection and sequence editing

**Figure 1:**
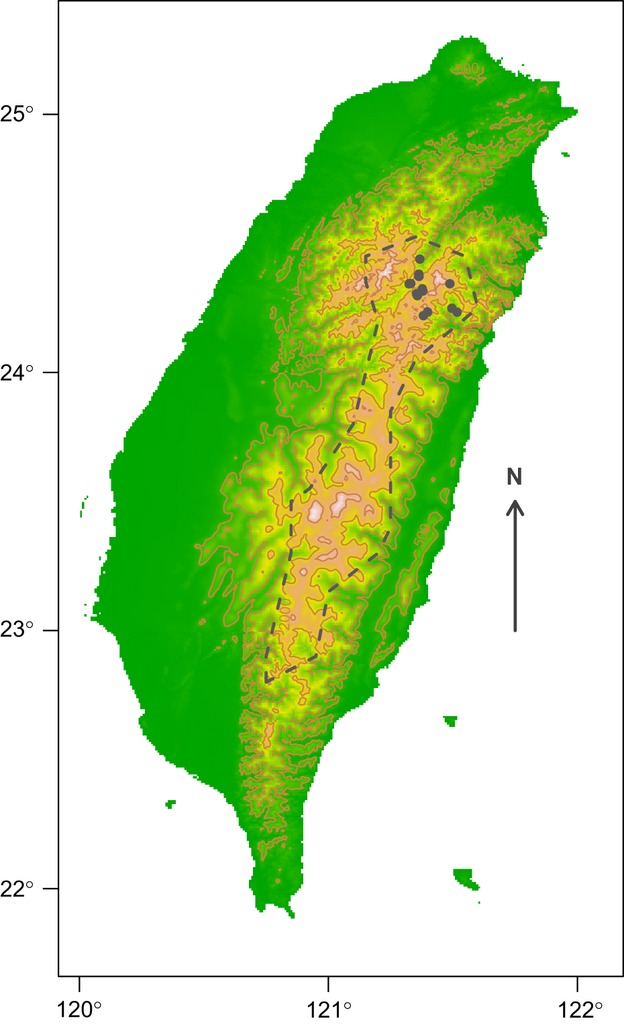
Relief map of Taiwan showing the sampling locations (black dots). The approximate distribution range of *Picea morrisonicola* is given by the dashed line and is based on the distribution given in Guan et al. 2009;. Relief data come from the Global Land 1-km Base Elevation Project (GLOBE Task Team et al. 1999). Contours correspond to altitudes of 500, 1000, 2000, and 3000 meters above sea level.

*Picea morrisonicola* occurs in sites distributed over the central mountain range of Taiwan at altitudes of 2300–3000 m (Guan et al. 2009). Seeds of *P. morrisonicola* were collected from 15 different trees growing in the northern part of this area (Fig. 1 and Table S1). Relief data for Taiwan were obtained from the Global Land 1-km Base Elevation Project (GLOBE Task Team et al. 1999). Fifteen nuclear loci (4CL, GI2, GI4, GI6, PCH, SE1107, SE1390, SE1464, SE1427, SE6, EBS, M002, SB16, SB29, and SB62) were used for sequencing. Eight loci were amplified using primers designed in previous studies (4CL, PCH, M002: Li et al. 2010; EBS, SE1390: Heuertz et al. 2006; SB16, SB29, SB62: Chen et al. 2010), whereas four loci were amplified using loci designed from an expressed sequence tag (EST) survey of Norway spruce individuals (SE1107, SE1464, SE1427, SE6: I. Ivanissevich and M. Morgante, unpubl. data, but see also Heuertz et al. 2006). The primers for loci GI2, GI4, and GI6 were designed based on the cDNA for the circadian clock gene *Gigantea* (N. Gyllenstrand, A. Karlgren, D. Clapham, A. Hall, K. Holm, P. D. Gould, T. Källman & U. Lagercrantz, unpubl. ms.). The loci were chosen to be single or low copy genes. DNA was extracted from the haploid megagametophyte tissue of one seed per individual using Qiagen plant DNA extraction kit (Hilden, Germany). All polymerase chain reaction (PCR) reactions were made using high-fidelity (HF) proof-reading Phusion enzyme in HF buffer (Finzymes, Espoo, Finland) and according to the instructions of the manufacturer. The PCR products were purified using Exo-SAP IT and sequenced on ABI3730 at Macrogen (Seoul, Korea) using both forward and reverse primers and in the rare cases where quality was not satisfactory we performed additional sequence reactions. Sequences were base called, visually inspected, and edited using the software suite Phred, Phrap, and Consed (Ewing et al. 1998; Ewing and Green 1998; Gordon et al. 1998). Only sequences with a Phred quality score above 20 were retained for further analysis. For the between-species analyses, eight loci (4CL, EBS, M002, PCH, SB16, SB29, SB62, and SE1390) for *P. likiangensis*, *P. purpurea*, *P. schrenkiana,* and *P. wilsonii* were taken from a previous study (Li et al. 2010) for the structure analysis, with a further six loci sequenced in *P. wilsonii* (GI2, GI4, GI6, SE6, SE1107, and SE1464) to supplement the coalescent-based analysis. Although none of the primers was developed specifically for *P. morrisonicola*, these primers have been used successfully in a number of studies on *Picea* species before. The fact that linkage decays fast in *P. abies* (Heuertz et al. 2006) supports the prerequisite that loci are physically unlinked. Seeds were sampled from 4, 6, 6 and 15 populations of *P. schrenkiana*, *P. wilsonii*, *P. purpurea,* and *P. likiangensis*, respectively, with a total of 23–80 individuals per species. All sequences have been archived in the data repository DRYAD with doi:10.5061/dryad.rm3n1.). The summary statistics Watterson's theta (θ_W_), Fay and Wu's theta (θ_H_), pairwise nucleotide difference (π), Tajima's D (*D*), Fay and Wu's non-normalized *H* (*H*), Zeng's normalized *H* (*Z*), and haplotypic diversity (*H*_e_) were calculated using the program EggLib (de Mita and Siol 2012). Significance of Tajima's D and Fay and Wu's *H* was calculated using the *libsequence* module *compute* (Thornton 2003).

### Genetic structure

To analyze the relationship of *P. morrisonicola* with the four mainland species (*P. likiangensis*, *P. purpurea*, *P. schrenkiana,* and *P. wilsonii*), we first used the software *Structure v. 2. 3. 2* (Pritchard et al. 2000). It uses unlinked markers to find the optimal number of clusters (*K*) in a data set. A total of 192 single-nucleotide polymorphisms (SNP) were recorded over the 10 loci where data were available for all five species. As markers that are extremely close together can interfere with the analysis, only SNPs that were situated at least 50 bp apart were retained, resulting in a final data set of 62 SNPs from eight loci. In total, 173 individuals from the five species were included in this analysis, and the number of successfully sequenced individuals per locus and species was 24–58 in *P. likiangensis*, 5–14 in *P. morrisonicola*, 10–22 in *P. purpurea*, 11–20 in *P. schrenkiana,* and 12–29 in *P. wilsonii*.

The admixture model with correlated allele frequencies was used. The LOCPRIOR function in Structure was employed, which uses information on sampling location of each indiviual to enhance the clustering. The results were also double checked with the independent frequency model. Burn-in length was set to 10,000 and the number of iterations after burn-in was 100,000. *Structure* was set to evaluate *K* = 1 through *K* = 10, and the results from 10 runs of each value of *K* were averaged. To find the optimal *K*, the average estimated likelihood of data was considered, as well as the summary plots and Δ*K* calculated as in Evanno et al. (2005).

### Estimate of divergence between *P. morrisonicola* and *P. wilsonii* using MIMAR

Based on the results from the *Structure* analysis that *P. wilsonii* is the closest relative to *P. morrisonicola* among the four species studied, we estimated divergence between *P. morrisonicola* and *P. wilsonii* with MIMAR (Becquet and Przeworski 2007, 2009)***.*** MIMAR is a program that estimates five demographic parameters in an IM model with two populations: the population mutation rate per base pair per generation for the ancestral population (θ_A_) and for the two descendant populations (θ_1_ and θ_2_), the time in generations since the split (*T*) and the symmetrical migration rate (*m*). The data are summarized by dividing the segregating sites into categories depending on their presence or absence in each of the populations. Then genealogies are simulated and subsequently accepted or rejected by a Markov chain Monte Carlo algorithm to give posterior distributions of the parameters of interest.

Outgroup sequences were taken from *Picea breweriana* (Chen et al. 2010) in most cases and from *P. schrenkiana* (Li et al. 2010) in the few loci where *P. breweriana* sequences were ambiguous or missing. The population mutation rate was set to 2.5 × 10^−8^ per site per generation, which is taken from the mean value of the upper and lower bounds of the estimate of the mutation rate per site and per year in the genus *Pinus* (0.7–1.31 × 10^−9^ site per year; Willyard et al. 2007) and *Picea* (0.6–1.1 × 10^−9^ per site per year; Chen et al. 2012) and adjusted to an assumed generation time comprised between 25 and 50 years (Brown et al. 2004; Petit and Hampe 2006). However, it should be noted that estimated mutation rates vary between species in the literature, and that a study on *Picea* found a somewhat lower mutation rate, although based on a much smaller number of loci (Bouillé and Bousquet 2005). In general, estimates of divergence time are sensitive to the given mutation rate and generation time and should therefore be interpreted with caution. After exploratory runs, the following uniform prior bounds were chosen: θ_1_∼*U*(0, 0.007), θ_2_∼*U*(0, 0.002), θ_A_∼*U*(0, 0.015), *T*∼*U*(0, 500,000), and ln(*m*) ∼*U*(−5, 1). The number of genealogies generated per step was increased to 50 as it was found to improve the acceptance rate considerably. Twenty million steps were performed after a burn-in of 2 million steps. The final analysis was done two times using different random seeds.

MIMARgof, a goodness-of-fit test, which is included in the MIMAR package, was performed on the results. It generates samples of summary statistics given the parameters estimated by MIMAR, and compares the distribution of the simulated samples with the observed values from the data set. This allows one to test whether the observed value falls within the range of the simulated values.

### Within-species ABC models

**Figure 2:**
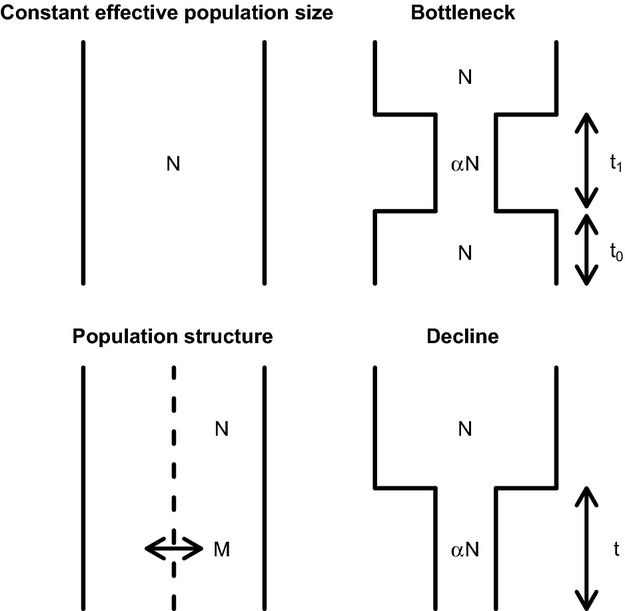
Within-population models of demography considered for ABC analysis, where *N* is the effective population size, α scales the effective population size during bottlenecks and declines, *M* is the population-scaled mutation rate (4*N*m), and *t*, *t*_1_, and *t*_0_ are the coalescent times of the decline and bottleneck events (measured in 4*N* generations).

To understand the demographic history of *P. morrisonicola*, four simple within-species demographic models (Fig. 2) were fit to the observed data. Three of these were chosen to reflect the observed positive Tajima's D value (0.28), which suggests a slight excess of intermediate variants. The population-scaled mutation and recombination rates are consistent across each of the models and are defined as θ = 4*N*μ and ρ = 4*N*r, respectively, where *N* is the effective population size, μ is the mutation rate per site, and *r* is the recombination frequency. Changes in the effective population size for the instantaneous bottleneck and population decline models are given by the parameter α, which represents the relative effective population size. For the instantaneous bottleneck model, a population of effective population size *N* experiences at *t*_0_ = *t* coalescent time units (where coalescent time units are measured in 4*N* generations) in the past a reduction in effective population size to α*N*, that persists for *t*_1_ *=* 0.2 coalescent time units, before returning to the original effective population size. We chose to fix the duration of the bottleneck as the severity and duration parameters are confounded in the model. In the population decline model, the effective population size changes from α*N* to *N t* coalescent time units in the past. A structured population model was also considered where two populations experience a symmetrical population-scaled migration rate *M* = 4*N*m, where *m* is the instantaneous migration rate. These models were compared with each other and against a null model with constant effective population size, consisting of just the two parameters θ and ρ. The priors for the model parameters were uniformly distributed as θ∼*U*(0, 0.01), ρ∼*U*(0, 0.02), *t*∼*U*(0, 1.5), *M*∼*U*(0.05, 1), α∼(0, 1.5). A total of 11 summary statistics (Table S2) were chosen to simulate parameters for each model using the above prior distributions. Waterson's theta, the mean and standard deviation of the average number pairwise differences (pi), haplotype diversity, and Tajima's D were used for model choice and parameter estimation based on the conclusion of previous studies into the performance of different sets of summary statistics (e.g., Clotault et al. 2012; Hickerson et al. 2006; Li and Jakobsson 2012; M. Stocks, M. Siol, M. Lascoux, S. de Mita, unpubl. ms.).

### Between-species ABC models

**Figure 3:**
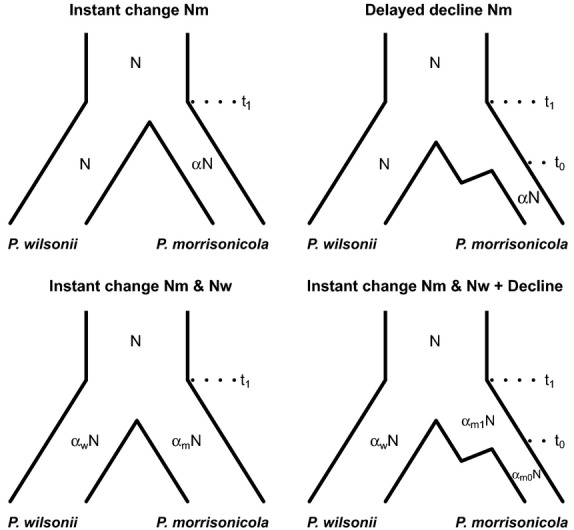
Between-population models compared in the ABC analysis, where *N* is the effective population size, α, α_m_, α_w_, α_m1_, and α_m0_ scale the effective population sizes during bottlenecks and declines, and *t*_1_ and *t*_0_ are the coalescent times of the divergence and decline events (measured in 4*N* generations).

MIMAR implements a split model for *P. wilsonii* and *P. morrisonicola* with and without migration, but assumes that effective population sizes remain constant through time and this could lead to problems inferring the correct divergence time. To test the robustness of the results obtained with MIMAR we therefore used ABC to compare the simple split model evaluated in MIMAR with more complex demographic models. Four between-species coalescent models were set-up based on a simple split model (Fig. 3). In the simplest scenario, an ancestral population of effective population size *N* split into two populations *t* coalescent time units in the past (measured in 4*N* generations). Due to the low diversity and positive Tajima's D in *P*. *morrisonicola*, we tested models that allow the effective population size of *P*. *morrisonicola* to vary. Two such scenarios are considered, an instant change model under which the effective population size of *P*. *morrisonicola* is reduced at divergence time (*IC*_*m*_), and another where the decline in effective population size is delayed until some time point between divergence time and the present day (*D*_*m*_). A model was also considered where the effective population sizes of both descendant populations went through an instantaneous change at divergence time (*IC*_*mw*_), and another similar model but with an additional decline in the effective population size of *P*. *morrisonicola* after divergence (*IC*_*mw*_ *+* *D*_*m*_). A simple split model was also considered where the effective population size remains constant throughout the coalescent process, but this model received such low support in the model choice step that it was not considered further (data not shown).

Each model is parameterized by the population-scaled mutation and recombination rates (θ = 4*N*μ and ρ = 4*N*r, respectively). Changes in the effective population size (α, αm, αw, αm0, and αm1) are estimated relative to *N* and the time since population divergence (*t*_1_) or a decline in *P. morrisonicola* (*t*_0_) is estimated in coalescent time units (measured in 4*N* generations). A set of 29 summary statistics were calculated when simulating parameters under each of the models (Table S2). The joint site frequency spectrum is the basic information on which the inference is based and can be summarized in various ways. We used Wakeley and Hey (1997) summary statistics as well as Tajima's D (calculated within Taiwan spruce) which has been shown to be informative in a number of studies investigating inference in ABC (e.g., Hickerson et al. 2006; Clotault et al. 2012; Li and Jakobsson 2012; M. Stocks, M. Siol, M. Lascoux, S. de Mita, unpubl. ms.). The priors were uniformly distributed with bounds θ∼*U*(0, 0.01), ρ:∼*U*(0, 0.02), α, αm, αw, αm0, αm1∼*U*(0, 1.5), and *t*_1,_*t*_1_∼*U*(0, 1.5).

### ABC parameter estimation, model choice, and posterior predictive simulations

For each model, 10^6^ simulations were performed and parameter estimation was performed using the ABC method of Beaumont et al. (2002) with log transformation of parameters and a tolerance of 0.001. We chose to produce a large number of simulations so that we can use a fairly strict tolerance, while sampling enough points from the posterior distribution to allow us to make inferences. This choice is based on a number of studies that have investigated in more detail the effect that different tolerances have on ABC inference (Hamilton et al. 2005; Li and Jakobsson 2012; Stocks et al. unpubl. ms.). Model choice was performed according to the method implemented in Fagundes et al. (2007), with Bayes factors calculated as the ratio of the marginal likelihoods of the competing models, *p*(*y|M*_1_)/*p*(*y|M*_0_). A Bayes factor of 3 was considered high enough to reject model *M*_0_ in favor of model *M*_1_ (Kass and Raftery 1995). For the most probable models of the within- and between-species analyses, 1000 parameter values were sampled from the posterior predictive distribution and were used to simulate summary statistics for comparison with the observed data. To assess the power and false-positive rate of ABC model choice, 100 simulated data sets were generated for each of the models. Each data set was simulated with the same number of samples and loci as that in the observed data. Model choice was then performed on each of the simulated data sets to establish the power and the false-positive rate of ABC. All ABC analyses, coalescent simulations, and summary statistic calculations for the ABC analyses were performed using the package *EggLib* (de Mita and Siol 2012).

## Results

### Nucleotide diversity and basic summary statistics

Seven of the 15 genes sequenced in *P. morrisonicola* were completely monomorphic. Estimates of total nucleotide diversity, Watterson's theta (θ_W_), and the average number of pairwise nucleotide differences (π) are low (Table 1). Averaged across loci, θ_W_*,* and π give values of 0.00147 and 0.00146 per base pair, respectively. This suggests that occurrences of low- and intermediate-frequency variants are in line with standard neutral expectations; however, six of eight polymorphic loci show an excess of intermediate-frequency variants (i.e., π > θ_W_). This is reflected by a positive value of 0.281 for Tajima's D when averaged across loci. An excess of intermediate variants is indicative of evolutionary processes whereby the time to coalescence of the remaining two lineages is longer than would be expected under a neutral coalescent model of constant effective population size. On the contrary, an excess of high-frequency variants was indicated by negative values of the normalized (*Z*) and non-normalized (*H*) versions of Fay and Wu's *H*. Two loci (4CL and SE1427) show significant deviations in Fay and Wu's *H* (*H*) from a simple coalescent model of constant effective population without recombination. The locus SE1427 also exhibits a significant deviation of Tajima's D from standard neutral expectations. However, few loci deviated significantly from neutral expectations and patterns varied among these summary statistics, warranting more detailed coalescent-based analyses.

**Table 1:** Summary statistics per locus where *n* is the number of individuals, *L* is the length and *S* is the number of segregating sites. The statistics given are Watterson's θ (θ_W_), the average number of pairwise nucleotide differences (π), Fay and Wu's θ (θ_H_), Ta jima's D (*D*), Fay and Wu's non-normalized *H* (*H*), Zeng's normalized *H* (*Z*) and the haplotypic diversity (*H*_e_). For Ta jima's D (*D*) and Fay and Wu's *H* (*H*)

Locus	*n*	*L*	*S*	θ_*W*_	π	*O*_*h*_	*D*	*H*	*Z*	*H*_e_
4cl	8	604	8	0.00511	0.00361	0.01484	−1.4213	−6.7857*	−3.6674	0.75
GI2	9	1590	0	0	0	0	–	–	–	0
GI4	10	985	0	0	0	0	–	–	–	0
GI6	14	1215	0	0	0	0	–	–	–	0
PCH	10	605	1	0.00058	0.00077	0.00033	0.8198	0.2667	0.5826	0.4667
SE1107	12	426	0	0	0	0	–	–	–	0
SE1390	11	551	7	0.00444	0.00629	0.00784	1.6842	−0.8364	−0.5199	0.7091
SE1464	15	419	0	0	0	0	–	–	–	0
SE6	15	445	1	0.00069	0.00094	0.00034	0.7421	0.2667	0.6112	0.419
ebs	8	785	0	0	0	0	–	–	–	0
m002	9	518	1	0.00072	0.00076	0.00267	0.1565	−0.9722	−2.0986	0.3889
sbl6	13	804	9	0.00362	0.00519	0.00959	1.7028	−3.5256	−1.8449	0.4615
sb29	13	515	0	0	0	0	–	–	–	0
sb62	13	540	2	0.00119	0.00142	0.00043	0.5437	0.5385	0.8062	0.3846
SE1427	12	468	8	0.00566	0.00285	0.02745	−1.9834*	−11.5152*	−6.5351	0.1667
Average		698		0.00147	0.00146	0.00423	0.2805	−2.8204	−1.5832	0.2498

* indicates loci that deviate significantly from the standard neutral model without recombination.

### Genetic structure

**Figure 4:**
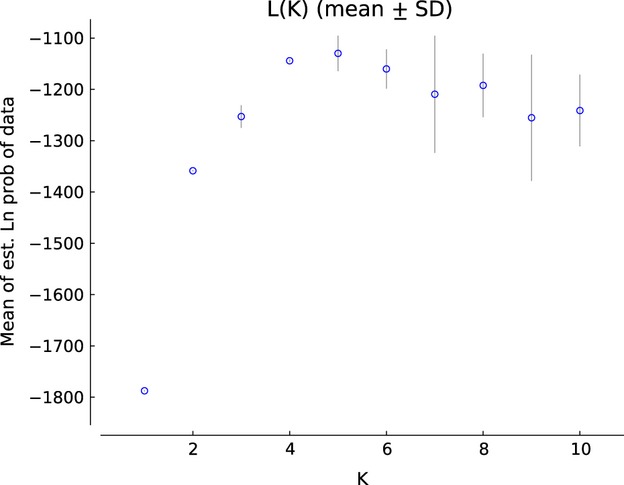
Likelihood and standard deviation for each value of *K* in the *Structure* analysis on the five species *Picea morrisonicola*, *Picea likiangensis*, *Picea purpurea*, *Picea schrenkiana,* and *Picea wilsonii*.

**Figure 5:**
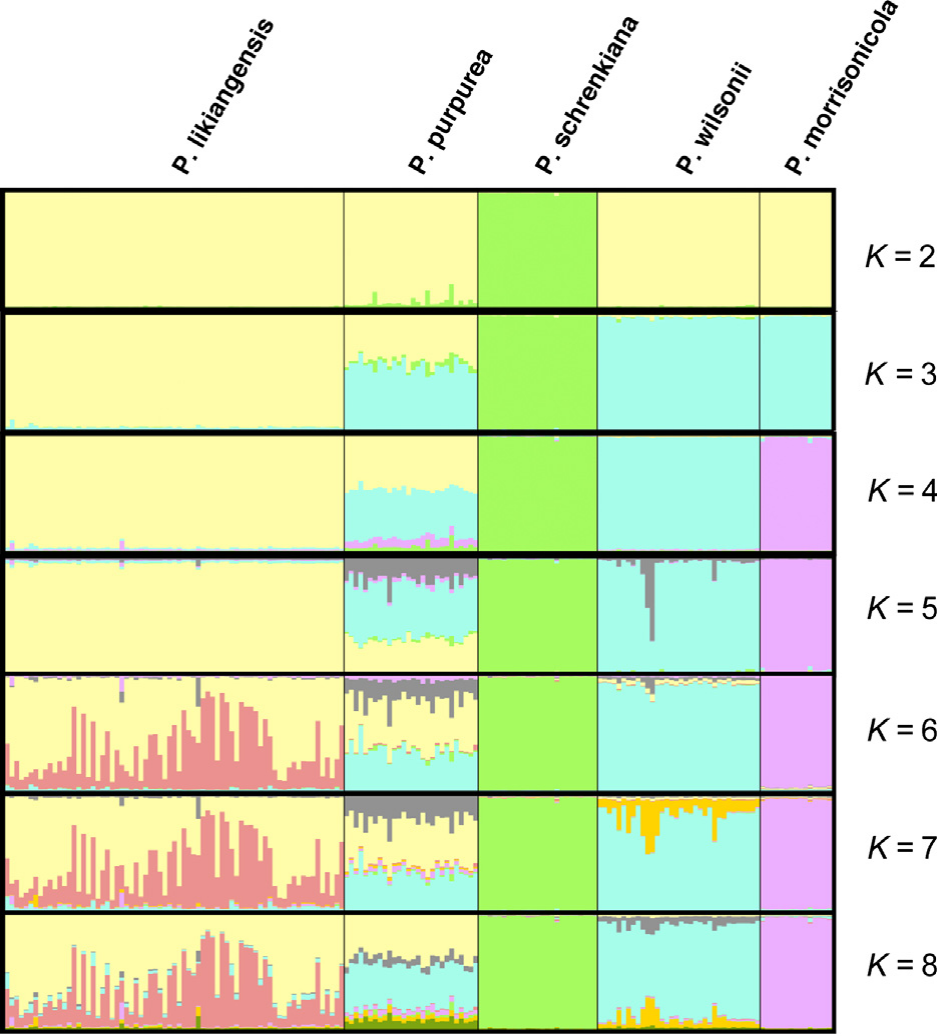
Ancestry estimates from *Structure* for *K* = 2 through *K* = 10 in the five species *Picea likiangensis* (P.l.), *Picea purpurea* (P.p.), *Picea schrenkiana* (P.s.), *Picea wilsonii* (P.w.), and *P. morrisonicola* (P.m.). Each *K* is represented by the *Structure* run that rendered the highest likelihood and shows the individual's estimated proportion of membership to each cluster.

The between-species clustering analysis suggests that *K* = 4 is the minimum number of clusters that captures the most important population structure (Fig. 4). Although *K* = 5 has a slightly higher probability, the curve starts to plateau at *K* = 4. At this level, all species except *P. purpurea* make up their own cluster (Fig. 5). Before that, at *K = 3*, *P. morrisonicola* clusters with *P. wilsonii*, suggesting that *P. morrisonicola* derived from the latter or that the two species have a common ancestor. The admixed nature of *P. purpurea* is in line with expectations as *P. purpurea* has previously been found to be a hybrid species between *P. wilsonii* and *P. likiangensis* (Li et al. 2010). The *K* calculation suggests *K* = 2 to be optimal, in which *P. schrenkiana* forms a cluster of its own (Table S3).

### Estimate of divergence between *P. morrisonicola* and *P. wilsonii* using MIMAR

Results from the analysis are shown in Table 2. *Picea wilsonii* and *P. morrisonicola* were found to have split about 160,000 generations ago, translating into 4 mya if a generation time of 25 years is assumed (5th–95th percentile 2.8–5.5 mya) or 8 mya if a generation time of 50 years is assumed (5th–95th percentile 5.5–11 mya). A low migration rate of 0.35 individuals per generation was detected. The population mutation rate, θ_2_, suggests that the size of the *P. morrisonicola* population after the split was around *N* = 5000 individuals, given μ = 2.5 × 10^−8^. The goodness-of-fit tests (Figure S3) show a good fit for some of the summary statistics, such as Tajima's D and the number of shared polymorphisms and fixed differences. Other summary statistics, including the number of segregating sites within *P. wilsonii* and the π calculated within *P. morrisonicola* (Table S6), show a significant departure suggesting that the retained model does not accommodate all aspects of the demography of the species. Possibly, this could be due to the presence of selection at some of the loci.

**Table 2:** Mode, 5% and 95% posterior density intervals of the two combined MIMAR runs on the split model between *Picea wilsonii* and *Picea morrisonicola*. The parameters are population mutation rate in *P. wilsonii* (θ_1_), *P. morrisonicola* (θ_2_) and the ancestral population (θ_A_), symmetrical migration rate (*M*) and split time in generations (*T*)

	θ_1_	θ_1_	θ_A_	*M*	*T*_gen_
Mode	0.005	0.0005	0.0008	0.35	160,000
5%	0.004	0.0003	0.0002	0.15	110,000
95%	0.006	0.001	0.004	0.61	220,000

### Within-species ABC

Table 3 shows Bayes factors and model probabilities for the four within-species demographic models applied to *P. morrisonicola*. The population decline model has the strongest support of all the models considered with a posterior probability of 0.5241. Bayes factors calculated between the population decline model and each of the competing models gave values of 8.7, 1.5, and 4.3 for the constant effective population size, bottleneck, and population structure models, respectively. Both the constant effective population size and structure models could be rejected in favor of the decline model, assuming a Bayes factor significance threshold of 3 (Kass and Raftery 1995). The bottleneck model cannot be rejected in favor of the population decline model. However, given that the Bayes factor favors a population decline we estimate parameters and perform posterior predictive simulations to assess the fit of this model to the data. Parameter values estimated under the population decline model are shown in Table 5 and Table S4: *P. morrisonicola*'s effective population size declined from around 124,000–1800 approximately 11,000 generations ago (assuming a per site and per generation mutation rate of 2.5 × 10^−8^). Assuming a generation time of 25 and 50 years, this would date the decline at approximately 270,000 years ago and 537,000 years ago, respectively.

**Table 3:** Bayes factors and model probabilities (in bold) amongst four competing within-species demographic scenarios. Bayes factors are calculated as the ratio of the marginal likelihoods of the two models under comparison: *p*(*y*|*M*_1_)/*p*(*y*|*M*_0_)

		*M*_0_			
		
		Constant Ne	Bottleneck	Decline	Structure
*M*_1_	Constant Ne	**0.1413**	0.2384	0.1148	1.0601
	Bottleneck	4.1948	**0.1993**	0.675	1.8329
	Decline	8.7087	1.4814	**0.5241**	4.2647
	Structure	0.9433	0.5456	0.2345	**0.1353**

The posterior predictive simulations under the population decline model are given in Figure S1. Tajima's D, haplotype diversity, and the standard deviation of π fit the observed data well. The relative site frequency spectrum also fit the data well, although the distributions of θ_H_ and *H* indicate that there is a relative excess of high-frequency–derived variants in the observed data compared to the posterior predictive distributions.

### Between-species ABC

Bayes factors and model probabilities for each of the four between-species models tested for *P. wilsonii* and *P. morrisonicola* are shown in Table 4. The delayed decline model (*D*_*m*_) shows the highest model probability (0.4512), and has Bayes' factors of 2.3, 3.7, and 1.8 when compared to the *IC*_*m*_*, IC*_*mw*_*,* and *IC*_*mw*_ *+ D*_*m*_ models, respectively. Neither the *IC*_*m*_ nor the *IC*_*mw*_ *+ D*_*m*_ models can be rejected (assuming a significant Bayes factor of 3), so we estimated the parameters of the most probable model (*D*_*m*_) and performed posterior predictive simulations to check the fit of the model to the data. Tables 5 and S5 show the parameters estimated under the *D*_*m*_ model and indicate that *P. wilsonii* and *P. morrisonicola* split from an ancestral population with an effective size of 44,000 approximately 45,000 generations in the past (assuming a per site and per generation mutation rate of 2.5 × 10^−8^). Within *P. morrisonicola* there was a reduction down to an effective population size of 4200 at around 15,000 generations in the past. Assuming a generation time of 25 years, this gives a divergence time of 1.1 million years and a population decline occurring around 374,000 years ago. Assuming a generation time of 50 years would double these estimates to give a divergence time of 2.2 million years and a population decline occurring 0.75 mya.

**Table 4:** Bayes factors and model probabilities (in bold) amongst four competing between-species demographic scenarios. Bayes factors are calculated as the ratio of the marginal likelihoods of the two models under comparison: *p*(*y*|*M*_1_)/*p*(*y*|*M*_0_)

		*M*_0_			
	
		*IC*_*m*_	*D*_*m*_	*IC*_*mw*_	*IC*_*mw*_ *+ D*_*m*_
*M*_1_	*Ic*_*m*_	**0.1828**	0.4276	1.4691	0.8051
	*D*_*m*_	2.3389	**0.4512**	3.6512	1.805
	*Ic*_*mw*_	0.6807	0.2739	**0.1285**	0.5209
	*IC*_*mw*_ *+ D*_*m*_	1.2422	0.554	1.9197	**0.2375**

**Table 5:** The effective population size and timing of demographic events for the within-species (ABCW) and between-species (ABCB) ABC analyses. The effective population sizes for the ancestral (*N*_1_ = θ/4μ) and present day (*N*_0_ = θ/4αμ)) populations are calculated using a mutation rate of 2.5 × 10^−8^. Coalescent time units, *t*, are measured in 4*N* generations, so the time in generations is given by *T* = 4*N t*. The time of divergence (*T*_1_) and of the population decline (*T*_0_) in years are calculated assuming a generation of 25 years. Point estimates were calculated using the mode of the posterior distribution

			Generations		Years	
				
	*N*_1_	*N*_0_	*T*_1_	*T*_0_	*T*_1_	*T*_0_
ABC_W_	123841	1783	**–**	10729	**–**	268236
ABC_B_	43737	4242	45056	14944	1126407	373598

Figure S2 compares statistics from 1000 posterior predictive simulations with the observed values. Overall, the fit of the model to the data is good. There are a number of statistics, such as D1 and *D*, that do not fit the data very well. However, between-species statistics such as the Wakeley and Hey statistics and *F*_ST_ fit the data well, which lends support to estimates of the divergence time inferred by the *D*_*m*_ model.

### Performance of ABC

There are two factors that need to be assessed due to the nature of the sampling. While population genetic studies typically require relatively few samples in order to capture important aspects of the genealogy, it is important to assess the power and false-positive rate of the model choice step of ABC. Furthermore, the choice of summary statistics for model comparison is critical to the output (Robert et al. 2011). There are a number of approaches (e.g., Joyce and Marjoram 2008; Wegmann et al. 2009; Nunes and Balding 2010; Aeschbacher et al. 2012; Fearnhead and Prangle 2012) that attempt to define sets of summary statistics that are sufficient *with regard to* the estimation of model parameters. It is, however, typically impossible to identify a finite-dimensional set of sufficient statistics (Sunnåker et al. 2013). Furthermore, these statistics will be sufficient with respect to model parameter estimation but not necessarily for distinguishing between models. Assessing the sufficiency of summary statistics for model comparison was not within the scope of this study, nonetheless we conducted a power analysis to assess whether the summary statistics chosen were informative for the models tested here. Table 6 shows the power (proportion of data sets simulated under the decline model where the null model is correctly rejected) and the false-positive rate (proportion of data sets simulated under a null model that are incorrectly rejected in favor of the decline model) for each of the models of the within-species analysis. The power varies depending on the model of comparison, with 0.81, 0.3, and 0.95 for the constant *N*, bottleneck, and structure models, respectively. The patterns produced by the bottleneck and decline models in contemporary modern data are expected to be similar and this is represented in the relatively low power allowed for rejecting the bottleneck model.

**Table 6:** The power and the false positive rate (FPR) for each model for a dataset with the same number of samples and loci as that used in the study. Power is defined as the proportion of simulated datasets that correctly reject (Bayes factor ≥3) the alternative model in favor of the decline mode. The FPR is defined as the proportion of simulated datasets for which the alternative model is incorrectly rejected (simulated Bayes factor ≥3 or ≥the observed Bayes factor) in favor of the decline model. The Bayes factors for the observed data, calculated for the given model against the decline model, are given in parentheses

	Constant Ne (8.71)	Bottleneck (1.48)	Structure (4.27)
Power	0.81	0.3	0.95
FPR_≥3_	0.07	0.01	0
FPR_≥obs_	0	0.12	0

However, the false-positive rate is relatively low. The constant *N* model has the highest false-positive rate (0.07), with the bottleneck model (0.01) and the structure model (0) having rates lower than 5%. Given that the Bayes factor for the decline model was not always greater than 3, it is also of interest to look at the proportion of simulated data sets that yield a Bayes factor greater than that calculated from the observed data. Among the simulated data sets for the constant *N* and structure models, there were no simulations for which the Bayes factor exceeded the observed Bayes factor. For the bottleneck model, although the false-positive rate was still low, 12% of the simulated data sets exceeded the observed Bayes factor.

## Discussion

The range and distribution of Taiwan spruce has changed with its surrounding environment and we aimed to look at the impact this change has had on genetic diversity. The foremost aims of this population genetic study of the endemic Taiwan spruce were to: (1) assess the genetic diversity in *P. morrisonicola* and compare it with the diversity of other spruce species of limited distributions, (2) identify the closest relative of *P. morrisonicola* and estimate the divergence time between these two species, and (3) quantify the impact of climate change on effective population sizes and assess the reliability of results obtained from a small sample. We find that *P. morrisonicola* is a species with a comparably low level of genetic diversity that went through a steep decline in its effective population size after splitting from *P. wilsonii* a few million years ago.

### Genetic diversity

Our survey of nucleotide diversity indicates that *P. morrisonicola* is among the less genetically diverse spruce species with a nucleotide diversity of the same order of magnitude to that observed in other marginal spruce species such as *P. breweriana* (π_s_ = 0.00,200; Chen et al. 2010) which grows in small scattered montane populations in Northern California, or *P. schrenkiana* (π_s_ = 0.00,258; Li et al. 2010), found in slightly larger populations in the Tian Shan mountain range at the northwestern border of China. Similar results have also been found in the Chihuahua spruce (*Picea chihuahuana)*, which shares several features with *P. morrisonicola*. *P. chihuahuana,* the southernmost spruce species of the American continent, is a relict species endemic to the montane forests of Mexico. In this species, very few chlorotypes and mitotypes were found, and the diversity within populations was low (*H* = 0.415 for chlorotypes and *H* = 0 for mitotypes). Two distinct mitotypes were found, which is interpreted as suggesting that the modern populations of this species originated from two ancestral populations in the near past after a bottleneck (Jaramillo-Correa et al. 2006). Although evidence is still limited, low genetic diversity might be common among peripheral populations in areas affected by glacial events. A richer sampling scheme, that covers a greater part of the species range, would no doubt lead to a more accurate estimate of the species nucleotide diversity. However, because population differentiation tends to be limited in forest trees due to extensive gene flow (Kremer et al. 2012) we do not believe that a larger sample size would significantly alter our conclusion.

### Origin and relationship to Taiwan geological history

*Picea morrisonicola* is a well-defined and distinct species and its closest relative among the four spruce species examined is *P. wilsonii*, as shown by the *Structure* analysis (Fig. 5). In contrast to the ancestry estimate plot (Fig. 5) and the calculated likelihoods for each *K* (Fig. 4), the Δ*K* analysis (Table S3) suggested only two clusters, in which *P. morrisonicola* would belong to the same cluster as all the other species examined except for the most geographically distant one, *P. schrenkiana*. However, Δ*K* is in some cases prone to underestimate the true *K*, for example, when differentiation between populations is not strong (Waples and Gaggiotti 2006). Based on other studies in conifers and the large number of shared polymorphisms observed even between species on different continents (Chen et al. 2010), we may indeed not expect very strong population structure in this case. Instead, *K = 4* can be justified from a biological point of view. Furthermore, our result suggesting that *P. wilsonii* and *P. morrisonicola* derive from the same common ancestor is corroborated by the multilocus phylogenetic analysis of the complete *Picea* genus that also place *P. morrisonicola* in the *P.wilsonii* clade (J. Liu, pers. comm.).

Assuming a generation time of 25–50 years, the split time between *P. wilsonii* and *P. morrisonicola* was estimated to be 4–8 million years by MIMAR and 1.1–2.2 million years by the *D*_*m*_ model of ABC. The latter estimates are close to those obtained in *Taxus* (Gao et al. 2007) and *Taiwania* (Chou et al. 2011), which colonized Taiwan 1.1 and 3.3 mya, respectively, but the former is significantly larger. The discrepancy between the estimates of divergence for the two methods can in the most part be attributed to differences in the models. The *D*_*m*_ model delays a reduction in the effective population size for a few million years compared to the scenario modeled in MIMAR. Also, the IM model implemented in MIMAR allows for gene flow after divergence, which was not taken into account in the between-species ABC models. Both should lead to a longer divergence time in MIMAR. Additionally, even if, as pointed out by a reviewer, these time estimates should be taken with a grain of salt due to their reliance on unwarranted assumptions about mutation rate and generation time, we note that they correspond well to either the time of formation of modern Taiwan, 4–5 mya, or the colonization by other conifer species, 1–3 mya.

Our data lend support to a scenario whereby *P. morrisonicola* evolved when migrants from the *P. wilsonii* clade reached Taiwan after the island was formed. This was during or just before the Pleistocene ice ages began (2.6 mya), and although Taiwan was never under ice cover, temperatures were lower than today. During the Pleistocene, repeated cycles of glacials and interglacials took place, whereby the population size of *P. morrisonicola* likely dropped considerably. Accordingly, a severe population decrease 0.37–0.74 mya was found by the *D*_*m*_ model of ABC. When temperatures started to rise again after the last glacial event, 11,800 years ago, *P. morrisonicola* may have begun retreating to the higher altitudes of central Taiwan, resulting in the population distribution seen today.

### Historical demography

We find evidence that the effective population size is smaller today than it was in the past. ABC analysis favors a within-species scenario where a 98.6% reduction in the effective population size occurs 11,000 generations in the past. This represents a drastic reduction in the effective population size, but may also explain why we are able to detect it. Due to the nature of our questions and the types of analyses used, our data collection focused more strongly on obtaining more loci as opposed to sampling more individuals. It is often the case that only limited samples are available and this can be a limiting factor in the estimation of range-wide genetic diversity, but may be much less problematic for inferences of past demographic events if a decent number of loci are available. Many of the most innovative population genetic studies carried out in *Drosophila* did not involve samples of X chromosomes from more than 10–20 individuals (e.g., Thornton and Andolfatto 2006; Singh et al. (2013)). *Drosophila* species generally have a very high level of genetic polymorphism and this may not be the case in other species. It was therefore important to assess how good our inferences could be based on small samples in a less variable species. Accordingly, our simulations of statistical power indicated that the ability to correctly reject a null model (i.e., constant size, bottleneck, or structure models) in favor of the decline model is low in small, genetically less diverse data sets. However, the false-positive rate, measured as the proportion of data sets for which the null model is incorrectly rejected, is also low. Overall, this means that while it is hard to reject a null hypothesis with a small sample size, a high Bayes factor is unlikely to be a false positive and instead represents a strong signal in the data.

### Conservation

Pollen analyses have previously shown that *Picea* was far more prevalent between 50,000 and 60,000 years ago (Tsukada 1966). The reduction in the range of Taiwan spruce and its retreat to higher altitudes has coincided with a general increase in temperature in Taiwan. Taiwan spruce, therefore, represents an excellent study species for assessing the long-term impact of climate change on genetic diversity. Although it is difficult to attribute any genetic changes with environmental shifts specifically, levels of genetic diversity are greatly reduced in Taiwan spruce compared to boreal spruce species and more in line with other spruce species with restricted distributions. Additionally, the data are consistent with the effective population size being considerably larger in the past, suggesting that environmental factors can have a big impact on genetic diversity within populations.

Temperatures are predicted to increase by between 0.9°C and 2.7°C in the next 30 years compared to 1961–1990 average temperatures (Hsu and Chen 2002). With studies correlating temperature with growth and demonstrating that a warmer January is negatively correlated with height growth (Guan et al. 2009, 2012), it is certainly of interest to consider how a further increase in temperature would affect Taiwan spruce in the long term. Although *P. morrisonicola* has survived past climate fluctuations, the ability to adapt during future climate changes may not be preserved for two reasons (Hermisson and Pennings 2005). First, adaptations starting from standing genetic variation critically depend on the amount of genetic variation available, therefore the removal of standing genetic variation in Taiwan spruce will reduce the probability that an adaptive allele is present in the current population. Second, the failure of Taiwan Spruce to recover to its predecline effective population size will limit the influx of new beneficial mutations into the population, a portion of which will inevitably be lost due to genetic drift. However, these represent more long-term rather than short-term concerns as to the conservation status of Taiwan spruce. It is worth noting that *P. morrisonicola* is relatively well protected in Taiwan today since logging of natural forests for commercial purposes was banned in 1991 and the remaining forests are still used for recreational and other less invasive purposes (World Forest Institute 2001).

Finally, from a conservation perspective, it would be of interest to assess the genetic diversity and structure in other locations across the distribution range. As this study focused on only the northern part of the distribution range, some of the diversity may still be undiscovered.

## Supporting Information

**Table S1.** Dutch red lists used in this study.

**Table S2.** List of attributes.

**Figure S1.** Definitions of prevalence, correct classification, type I error and type II error probability.

**Figure S2.** Theoretical effect of prevalence, i.e., the number of declining species divided by the total number of species, of the species group and type I and type II error probabilities on correct classifications. The effect of three examples of combinations of type I and type II error probabilities are shown.
