# Supplementary figures and images for: Origin and demographic history of the endemic Taiwan spruce (Picea morrisonicola)
Source: Ecol Evol. 2013 Aug 15;3(10):3320–33. doi: 10.1002/ece3.698 (PMC3797480; doi:10.1002/ece3.698)

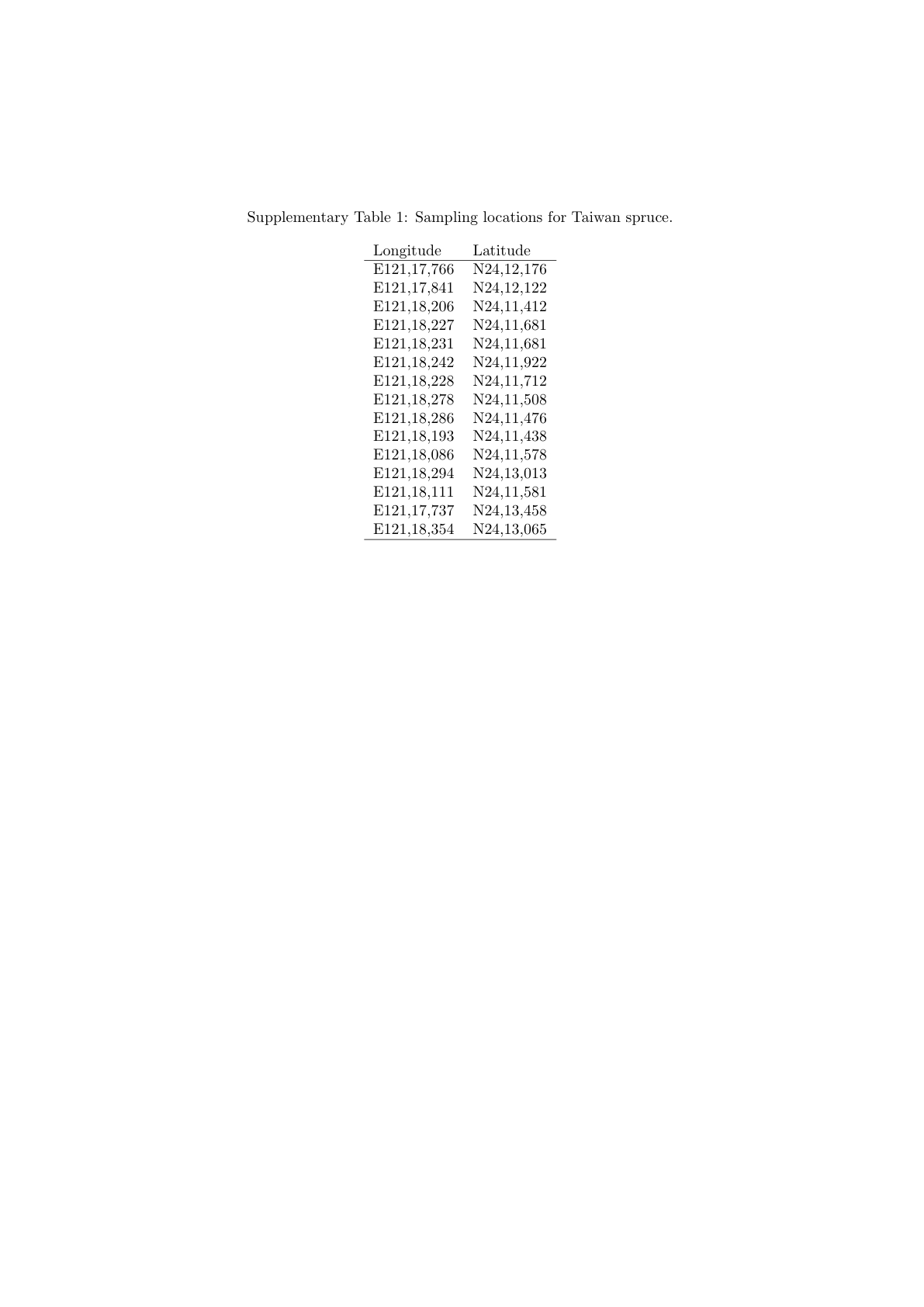

Supplement: Supplementary file 1 [file ece30003-3320-SD1.jpg]

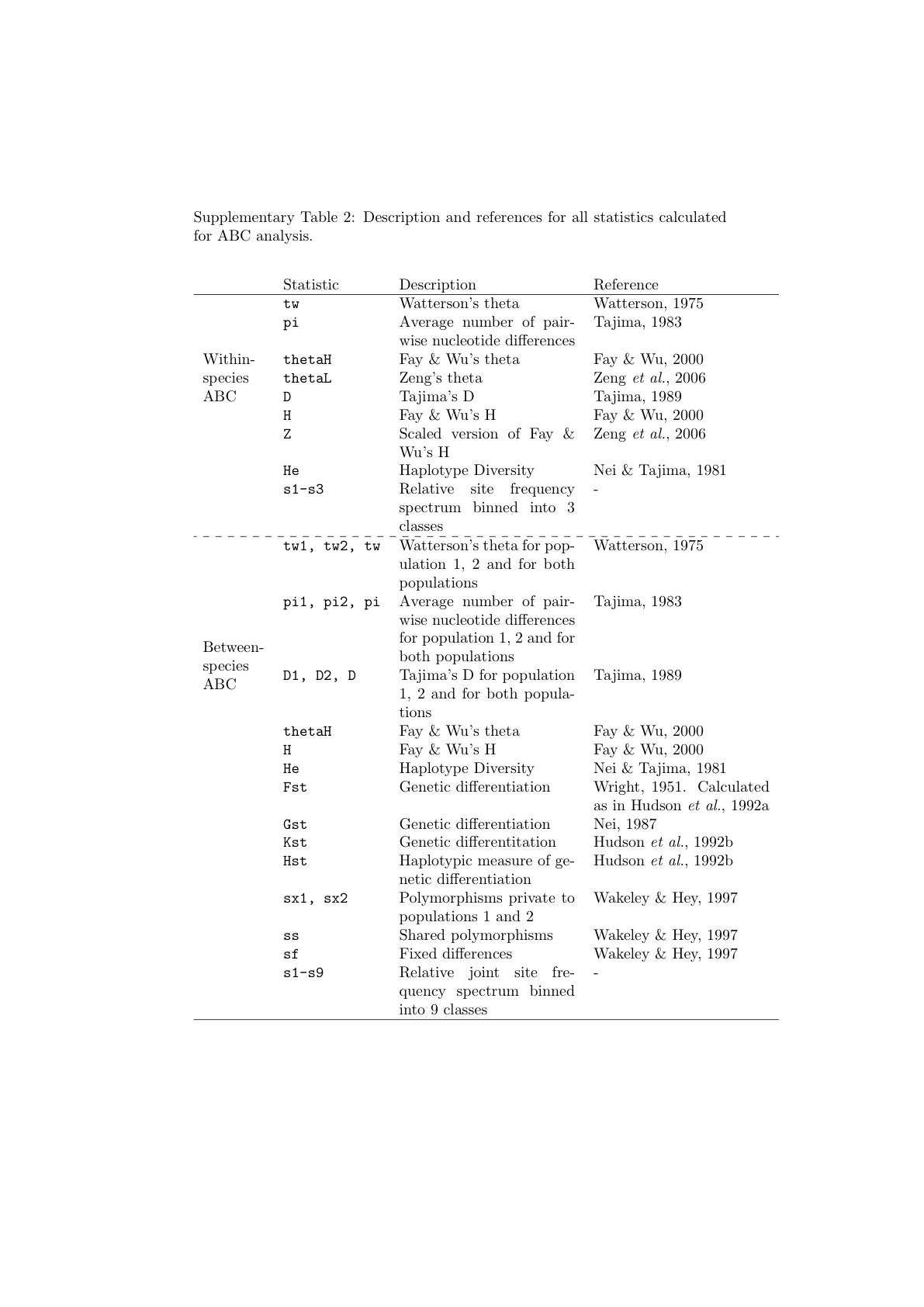

Supplement: Supplementary file 2 [file ece30003-3320-SD2.jpg]

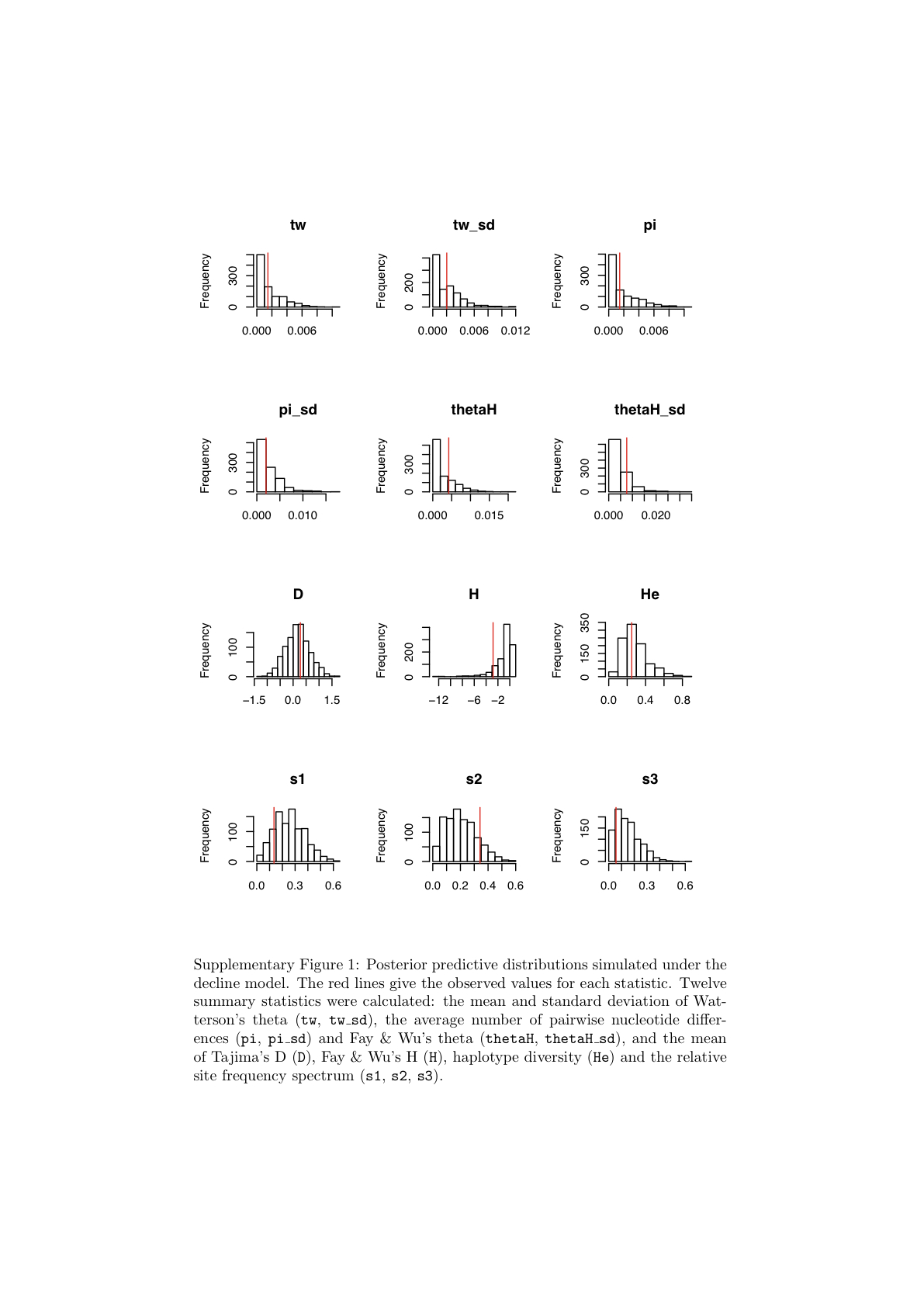

Supplement: Supplementary file 3 [file ece30003-3320-SD3.jpg]

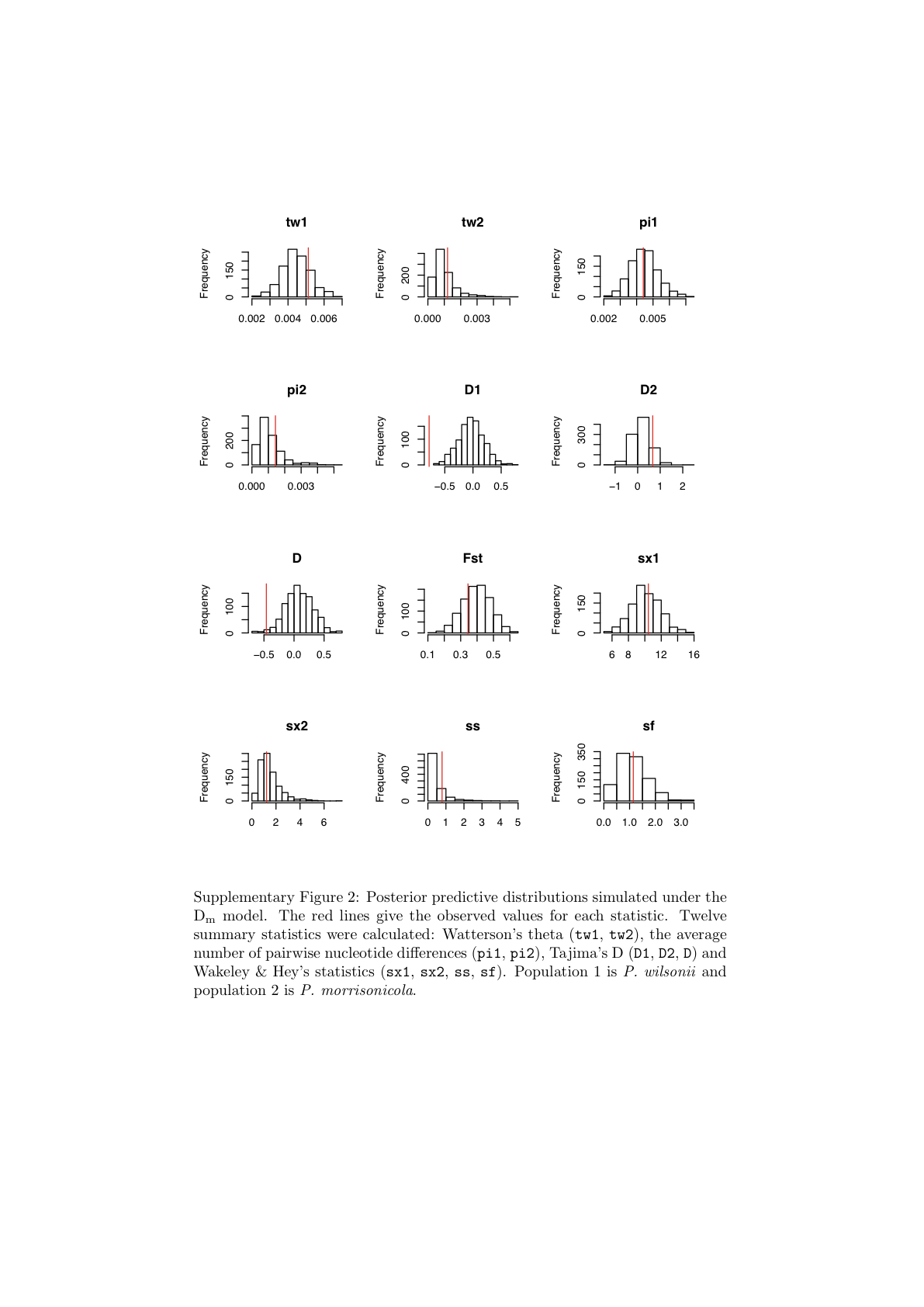

Supplement: Supplementary file 4 [file ece30003-3320-SD4.jpg]
